# Effects of low nitrogen on seedling growth, photosynthetic characteristics and antioxidant system of rice varieties with different nitrogen efficiencies

**DOI:** 10.1038/s41598-023-47260-z

**Published:** 2023-11-13

**Authors:** Zexin Qi, Fenglou Ling, Dongsheng Jia, Jingjing Cui, Zhian Zhang, Chen Xu, Lintian Yu, Chenglong Guan, Ye Wang, Mengru Zhang, Jiaqi Dou

**Affiliations:** 1 College of Agriculture, Jilin Agricultural University, Changchun, 130118 China; 2 Institute of Agricultural Resources and Environment Research, Jilin Academy of Agricultural Sciences (Northeast Agricultural Research Center of China), Changchun, 130033 China

**Keywords:** Physiology, Plant sciences

## Abstract

Nitrogen plays a significant role in influencing various physiological processes in plants, thereby impacting their ability to withstand abiotic stresses. This study used hydroponics to compare the effects of three nitrogen supply levels (1N, 1/2N and 1/4N) on the antioxidant capacity of rice varieties JJ88 (nitrogen efficient) and XN999 (nitrogen inefficient) with different nitrogen use efficiencies. The results show that compared with the XN999 variety, the JJ88 variety has stronger adaptability to low-nitrogen conditions, which is mainly reflected in the relatively small decrease in dry weight and net photosynthetic rate (Pn); In the early stage of low-nitrogen treatment (0–7 d), the ${\mathbf{O}}_{2}^{-}$ production rate, hydrogen peroxide (H_2_O_2_) and malondialdehyde (MDA) content of JJ88 variety increased relatively slightly, but the superoxide dismutase (SOD), peroxide The activity of enzyme (POD) and catalase (CAT) increased significantly; After low-nitrogen treatment, the ASA–GSH cycle enzyme activity of JJ88 variety was relatively high, and the dehydroascorbate reductase (DHAR) activity after 14 days of low-nitrogen treatment was higher than that of 1N treatment; The content of reduced ascorbic acid (ASA) in non-enzymatic antioxidants was lower than that of 1N treatment after 14 days of low nitrogen treatment; The contents of oxidized dehydroascorbic acid (DHA) and carotenoids (Car) were higher than those of 1N treatment after 21d and 14d of low nitrogen treatment respectively; The contents of reduced glutathione (GSH), oxidized glutathione (GSSG) and proline (Pro) showed a larger upward trend during the entire low-nitrogen treatment period. In summary, the JJ88 rice variety has a strong ability to regulate oxidative stress and osmotic damage under low nitrogen conditions. It can slow down plant damage by regulating antioxidant enzyme activity and antioxidant content. This provides a basis for achieving nitrogen reduction and efficiency improvement in rice and the breeding of nitrogen-efficient varieties.

## Introduction

Rice is a major cereal crop that plays an important role in global food production and security^1^. As the global population grows, so does its demand^2^. Currently, 3.5 billion people consume 748 million tons of rice, accounting for 15% of global nitrogen fertilizer use^3^. Assuming that the world’s population reaches 10 billion by 2050, nitrogen demand for rice production will increase by 44%^4^. With the acceleration of urbanization, the area of cultivated land continues to decrease. To increase rice production, productivity must be increased by increasing unit yields^5,6^. With crops utilizing only 30–40% of applied nitrogen, the remaining 60–70% of unused nitrogen in agriculture is causing serious environmental and health problems^7^. Unused soil nitrogen volatilizes into the air in the form of pollutants such as ammonia and nitrogen oxides, harming ecosystems through ozone depletion, eutrophication, and soil acidification^8,9^. In addition, it also increases the sensitivity of crops to lodging and diseases, limiting the improvement of rice yields. In order to reduce resource waste and pollution and achieve sustainable development, optimizing nitrogen utilization is crucial. This can currently be achieved by selecting or cultivating varieties with high nitrogen utilization efficiency in large-scale planting systems^10–12^.

In plants, photosynthesis is a key physiological process for growth and development, and its efficiency is affected by the amount of nitrogen applied^13,14^. The absorbed N is mainly used for photosynthesis^15^. Among them, photosynthesis plays an important role in the formation of crop yields, providing nearly 70% of the materials for crop yields^16^. Furthermore, gas exchange parameters showed significant correlations among traditional rice varieties^17,18^. Therefore, the function of these physiological traits directly affects plant growth and crop yield^19^. Research shows that nitrogen nutrition management is of great significance in delaying leaf senescence and extending photosynthesis time^20^. Yi et al.^21^ reported that increasing fertilization increased the leaf area index and the photosynthetic capacity of the canopy, thereby promoting biomass production. Zhou et al.^22^ have shown that chlorophyll content and net photosynthetic rate increase with the increase in nitrogen fertilizer application, green leaf area is maintained longer, and photosynthetic potential is higher. These are beneficial to rice photosynthesis, thereby increasing grain yield. Therefore, research on the low-nitrogen tolerance of rice under low-nitrogen conditions is of great significance for the breeding of nitrogen-efficient rice varieties.

In plants, stress can cause severe physiological and morphological effects. However, plant growth is inhibited by causing oxidative stress through the production of reactive oxygen species (ROS)^23^. Research shows that stress-induced oxidative stress is due to the production of ROS, such as ${\mathrm{O}}_{2}^{-}$ and H_2_O_2_^24,25^. These ROS are highly cytotoxic and can react with important biomolecules such as lipids, proteins, and nucleic acids, causing lipid peroxidation, membrane damage, and enzyme inactivation respectively, thereby producing toxic effects^26^. The main organelles that produce ROS in cells are chloroplasts, mitochondria, peroxisomes, exoplasts and endoplasmic reticulum^27^. To minimize and/or prevent the toxic effects of these damaging ROS, plants have evolved highly regulated enzymatic and non-enzymatic mechanisms to maintain a balance between ROS production and destruction to maintain cellular redox homeostasis^28^. The main reactive oxygen species scavenging enzymes are SOD, POD, CAT, APX, GPX and GR^29,30^. Non-enzymatic antioxidants include small molecular weight antioxidants such as alpha-tocopherol, Car, ASA, GSH, flavonoids and total phenols. Other non-enzymatic antioxidants, such as soluble sugars, trehalose, polyamines, proline, and glycine betaine, also help regulate ROS. Proline not only regulates permeability under stress, but also inhibits ROS, stabilizes ROS scavenging enzymes, and protects plant tissues from ROS damage^29^.

In recent years, the impact of nitrogen deficiency on plant antioxidants has attracted widespread attention^28^. It was reported that the SOD and CAT activities of tolerant ryegrass increased in roots and the CAT activity of susceptible ryegrass decreased in stems after 20 days of low nitrogen treatment^31^. Other studies have shown that nitrogen deficiency leads to an increase in MDA content and SOD activity in cannabis. Nitrogen supply and cultivar are related to the growth, physiology and nutrient accumulation of cannabis under hydroponic conditions^32^. Ahmad et al.^33^ found that increasing nitrogen application could enhance the SOD, CAT and POD activities of two sorghum varieties, thereby improving the morphological and physiological activity of sorghum. It can be seen that the antioxidant responses of different crops under low nitrogen conditions are very different, and there are also differences between different varieties of the same crop. Liao et al.^34^ reported that increased application of nitrogen fertilizer regulated the activities of SOD, POD and CAT. The MDA content of rice decreased under high nitrogen conditions, and there were differences among rice varieties of different genotypes. Antioxidant response is a dynamic change, and research on the antioxidant system of rice under low nitrogen conditions has not been reported. Therefore, this study analyzed the growth characteristics, photosynthetic characteristics, reactive oxygen metabolism, membrane lipid peroxidation level, antioxidant enzyme activity, ASA–GSH cycle enzyme activity and antioxidant content of rice varieties with different nitrogen efficiencies under continuous low nitrogen conditions. The research results can provide theoretical basis for nitrogen reduction and efficiency improvement and nitrogen fertilizer management in rice.

## Materials and methods

### Experiment material

Under low-nitrogen conditions at the rice seedling stage, 266 rice materials provided by the Rice Research Institute of Jilin Agricultural University were screened. According to the factor value and low nitrogen tolerance coefficient of each comprehensive index, based on the comprehensive index value (F) in the principal component analysis result, membership function analysis is used to calculate the membership value (U) of every principal component, and then based on the weight of each indicator Calculate the low nitrogen tolerance comprehensive score value (D) of each rice material. The larger the D value, the stronger the ability to tolerate low nitrogen. Variety screening method based on Yuan^35^. The Jijing 88 (JJ88) rice variety has a higher D value and has strong low-nitrogen tolerance, while the Xinong 999 (XN999) rice variety has a lower D value and has a weak low-nitrogen tolerance. The JJ88 rice variety is widely promoted in Jilin Province due to its similarities in growth period (mid-late maturing) with the XN999 rice variety. Therefore, these two rice varieties were chosen for the study.

### Experimental design and management

The experiment was conducted in the greenhouse of Jilin Agricultural University (43°48′37″N, 125°24′14″E) in 2022, using natural lighting. On May 10, the seeds were disinfected with 0.5% sodium hypochlorite for 10 min, and the disinfectant attached to the surface of the rice seeds was removed with distilled water and placed on a petri dish for germination. On May 13, the germinated seeds were sown on vermiculite, and 3 plants were sown in each hole. Grow in water until May 16, place the seedling trays on the seedling boxes, and the nutrient solution in each seedling box is 20 L. First use 1/2 concentration Kimura B nutrient solution to cultivate until May 19, and then use the improved Kimura B nutrient solution. After June 2, switch to Kimura B nutrient solution with different nitrogen concentrations (using NO_3_^−^ and NH_4_^+^ as nitrogen sources) for treatment. After 21 days of treatment, the rice grew to four leaves and one center. The nutrient solution was replaced every 3 days to maintain the pH. The concentration of H_2_PO_4_^−^, K^+^, Ca^2+^, Mg^2+^, Na^+^ and trace elements in the nutrient solution remained constant. Change, where Fe is replaced by Fe (EDTA-Na_2_).

Three nitrogen concentration treatments were set up, each treatment was repeated three times, and each replicate had 600 plants. The concentrations of NO_3_^−^ (1.6 mM) and NH_4_^+^ (1.6 mM) in the improved Kimura B nutrient solution are recorded as 1N, the concentrations of NO_3_^−^ (0.8 mM) and NH_4_^+^ (0.8 mM) are recorded as 1/2N, and the concentrations of NO_3_^−^ (0.4 mM) and NH_4_^+^ (0.4 mM) are recorded as 1/4N. Samples were taken at 0, 7, 14, and 21 days of treatment. Samples for enzyme activity measurement were immediately frozen in liquid nitrogen and stored in a − 80 °C refrigerator for subsequent analysis.

### Plant sampling and measurements

#### Growth indicators and photosynthetic parameters

After 21 days, three rice seedlings with basically the same growth status were selected from each treatment, and the rice plant height, leaf area, root number, above-ground dry weight, and root dry weight were measured.

After 21 days, a portable photosynthesis measurement system (Li-6400, LI-COR Inc., Lincoln, NE, USA) was used to measure the net photosynthesis of functional leaves (inverted leaves 3–4) at 8:00–11:00 am. Photosynthetic rate (Pn), stomatal conductance (Gs), transpiration rate (Tr) and intercellular carbon dioxide concentration (Ci), the light intensity in the leaf chamber was set to 1200 μmol·m^−2^ s^−1^. For each treatment, three rice seedlings with basically the same growth potential were selected. Water use efficiency (WUE) = Pn/Tr.

#### Enzyme activity and substance content

Fresh samples (0.30 g) were homogenized in an ice bath with 3 ml of PBS, and centrifuged at 8000 rpm for 15 min at 4 °C; the supernatant was used to determine SOD, POD, CAT activity and MDA content.

The SOD activity was determined using the nitro blue tetrazolium method. Absorbance was measured at 560 nm. One unit of SOD activity is defined as 50% inhibition of color reaction, expressed in U/g FW^36^.

Enzyme extract (50 μL) was added to the reaction solution containing 1 mL 0.3% H_2_O_2_, 0.95 mL 0.2% guaiacol, and 1 mL 50 mM sodium phosphate buffer (pH 7.0) to determine POD activity. Measure its absorbance at 470 nm. Unit POD activity is defined as the increase in absorbance by 1 per minute, and the unit is U/g FW^36^.

The enzyme extract (50 μL) was added to the reaction solution containing 1 mL 0.3% H_2_O_2_ and 1.95 mL sodium phosphate buffer to determine the CAT activity, and the absorbance was measured at 240 nm. The unit of CAT activity is defined as an increase in absorbance of 0.01/min, and the unit is U/g FW^36^.

The thiobarbituric acid method was used to determine the MDA content. React MDA and TBA, and record the absorbance of the reaction solution at 532, 600, and 450 nm. MDA content is expressed in μmol/g FW^36^.

The H_2_O_2_ content was measured according to the method of Zhang^37^.Weigh 2 g of fresh plant tissue, add 2 mL of acetone pre-cooled at 4 °C and a little quartz sand to grind it into a homogenate, centrifuge at 3000 r/min for 10 min, discard the residue, and use the supernatant for measurement. Take 1 mL of the sample extraction solution, add 0.1 mL of 5% titanium sulfate and 0.2 mL of concentrated ammonia. After a precipitate forms, centrifuge at 3000 r/min for 10 min, and discard the supernatant. The precipitate was washed repeatedly with acetone 3–5 times until the plant pigments were removed. Add 5 mL of 2 mol/L sulfuric acid to the washed precipitate. After it is completely dissolved, carefully transfer it to a 10 mL volumetric flask, rinse the centrifuge tube with a small amount of distilled water several times, combine the washing liquids, and adjust the volume to 10 mL. Scale, measure absorbance at 415 nm.

The ${\mathrm{O}}_{2}^{-}$ production rate was measured according to the method of Zhang^37^.Weigh 5 g of fresh plant material, add 10 mL of 50 mmol/L PBS with pH = 7.8, grind, centrifuge at 5000 g for 10 min, and take the supernatant. Take 1 mL of the above supernatant, add 0.9 mL of 50 mmol/L PBS, and 0.1 mL of hydroxylamine hydrochloride (replace the sample supernatant with PBS as a blank). After mixing, incubate in a constant temperature water bath at 25 °C for 30 min. Then take 1 mL of the culture solution and add 1 mL of p-aminobenzenesulfonic acid and 1 mL of α-naphthylamine respectively, incubate and react in a constant temperature water bath at 25 °C for 20 min, add 3 mL of n-butanol, and measure the absorbance at 530 nm.

The Car content was measured according to the method of Zhang^37^. Use the 80% acetone immersion method to measure the absorbance at 645 nm, 663 nm, and 470 nm.

APX activity was measured according to the method of Nakano and Asada^38^. Weigh 0.2 g of the plant sample into a pre-cooled mortar, and add 2 mL of pre-cooled extraction solution (50 mmol/L Na_2_HPO_4_-NaH_2_PO_4_ buffer, pH = 7.0, containing 1.0 mM ASA, 1.0 mM EDTA, 1% PVP, w/v) on an ice bath, grind it into a homogenate, and centrifuge at 12,000 r/min for 10 min at 4 °C. The supernatant is the APX crude extract. During the measurement, add 0.1 mL of enzyme extract solution to 2.9 mL of the reaction mixture (50 mM Na_2_HPO_4_–NaH_2_PO_4_ buffer, pH 7.0, containing 1.0 mM ascorbic acid and 2.5 mM H_2_O_2_). After mixing, monitor the change at 290 nm for 3 min.

GR activity was measured according to the method of Li^39^. Weigh 0.2 g of the plant sample into a pre-cooled mortar, and add 2 mL of pre-cooled extraction solution (50 mmol/L Na_2_HPO_4_–NaH_2_PO_4_ buffer, pH = 7.0, containing 1.0 mM EDTA, 1% PVP, w/v) Grind into a homogenate on an ice bath, and centrifuge at 12,000 r/min for 10 min at 4 °C. The supernatant is the GR crude extract. During the measurement, add 0.1 mL of enzyme extract solution to 2.9 mL of the reaction mixture (50 mM Na_2_HPO_4_-NaH_2_PO_4_ buffer, pH 7.8, containing 5 mM MgCl_2_ and 0.5 mM GSSG). After mixing, monitor the change at 340 nm for 5 min.

MDHAR activity was measured according to the method of Liu^40^. Weigh 0.2 g of the plant sample into a pre-cooled mortar, and add 2 mL of pre-cooled extraction solution (50 mmol/L Tris buffer, pH = 7.0, containing 0.3 M mannitol, 1.0 mM EDTA, 1% bovine serum albumin, w/v, 1% cysteine) was ground into a homogenate on an ice bath, and centrifuged at 12,000 r/min for 10 min at 4 °C. The supernatant was the MDHAR crude extract. When measuring, take 2.8 mL of the reaction mixture (100 mM Tris–HCl buffer, pH = 7.2, containing 1 mM ascorbic acid and 0.2 Mm ADH), add 0.1 mL of enzyme solution, mix and add 0.1 mL of 0.2U ascorbic acid oxidase (AO) Start the reaction and measure the absorbance at 340 nm, reading every 30 s.

DHAR activity was measured according to the method of Liu^40^. The extraction method of crude enzyme solution is the same as MDHAR. When measuring, take 2.8 mL of the reaction mixture (100 mM K_2_HPO_4_–KH_2_PO_4_ buffer, pH = 6.3, 1 mM dehydroascorbic acid) and add 0.1 mL of enzyme solution. After mixing, add 0.1 mL GSH to start the reaction. Measure the absorbance at 265 nm and read every 30 s.

The content of ASA and DHA was determined using the dipyridyl method, referring to the method of Aravind and Queva^41,42^. 0.2 g sample was added to 2 mL of 5% metaphosphoric acid in an ice bath and ground into a homogenate, centrifuged at 15000 g for 20 min, and the supernatant was used for determination. Determination of total ascorbic acid (ASA + DHA) content: Take 0.2 mL of supernatant in a centrifuge tube, add 0.2 mL of 150 mM PBS (pH = 7.4, containing 5 mM EDTA) and 0.1 mL of 10 mM DTT, vortex, and place at room temperature for 15 min. Add 0.1 mL of 0.5% *N*-ethylmaleimide. Shake well, then add 0. mL 10% TCA, 0.4 mL 44% H3PO4, 0.4 mL bipyridine (70% ethanol configuration) and 0.2 mL 3% FeCl3, and vortex. Place in a 37 °C water bath for 1 h, centrifuge to remove impurities, and measure the absorbance at 525 nm. The ASA content was measured according to the above steps, using distilled water instead of DTT, and the other steps remained unchanged. The DHA content is equal to the difference between the total ascorbic acid content and the ASA content.

The GSH and GSSG contents were measured using the DTNB cycle detection method, referring to the method of Cohn and Griffith^43,44^. Add 2 mL of 6% metaphosphoric acid to the sample in an ice bath to grind 0.2 g of sample into a homogenate, centrifuge at 12,000 g for 20 min, and use the supernatant for measurement. Determination of total glutathione (GSH + GSSG) content: Take 1.8 mL 100 mM PBS (pH7.8, containing 6 mM DTNB, 0.5 Mm NADPH), add 0.1 mL supernatant, and immediately add 0.1 mL 3 units of GR to start reaction, measure the absorbance at 412 nm, and read it every 30 s. Determine the GSSG content according to the above steps. The supernatant needs to be pretreated (0.1 mL of supernatant plus 50 μL of 2-vinylpyridine, react in the dark at 25 °C for 1 h), and the other steps remain unchanged. GSH content is the difference between total glutathione content and GSSG content.

The proline content was measured according to the method of Li^45^. Weigh 0.5 g of the plant sample, cut into pieces and put into a test tube with a stopper, add 5 mL of 3% sulfosalicylic acid, extract in a boiling water bath for 10 min, filter, and the filtrate will be tested. Take 2 mL of the filtrate and place it in a test tube, then add 2 mL of glacial acetic acid and 2 mL of ninhydrin reagent, cover and seal, and heat on a boiling water bath for 30 min. After cooling, add 5 mL of toluene to each sample, shake well for extraction, and let stand in the dark until complete stratification. Use a pipette to absorb the toluene layer into a cuvette, and measure the absorbance at 520 nm.

### Data analysis

Excel 2010 software was used for preliminary statistics and organization of data, and IBM SPSS Statistics 22.0 software was used for variance analysis. The least significant difference (LSD) test was used, *P* < 0.05. This data was created with Microsoft Office 2016. The R language software “gpairs” package was used for correlation analysis and drawing.

## Results

### Rice seedling growth

**Figure 1:**
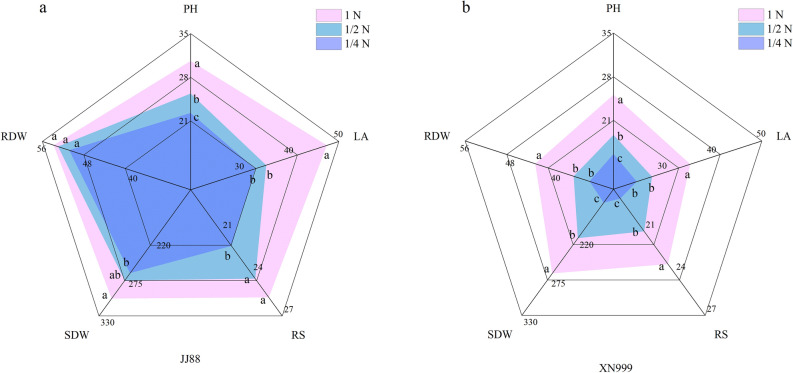
Effect of nitrogen deficiency on rice seedling growth (**a, b**). Each angle of the pentagon showed different traits, PH—plant height (cm), LA—leaf area (cm^2^/plant), RS—number of roots, SDW—aboveground dry weight (mg/plant), RDW—underground dry weight (mg/plant) .

Rice varieties with different nitrogen efficiencies showed obvious differences in seedling growth after low nitrogen treatment (Fig. 1). Under 1/2N treatment, the plant height and leaf area of JJ88 rice variety were significantly (*P* < 0.05) reduced by 17.09% and 25.81%, respectively. The plant height, leaf area, root number, aboveground dry weight and underground dry weight of the XN999 rice variety were significantly (*P* < 0.05) reduced by 25.26%, 21.80%, 10.00%, 18.62% and 15.39% (Fig. 1a). Under the 1/4N treatment, the plant height, leaf area, root number and above-ground dry weight of the JJ88 rice variety were significantly (*P* < 0.05) reduced by 27.22%, 30.48%, 14.29% and 11.89%. The number, aboveground dry weight and underground dry weight were significantly (*P* < 0.05) reduced by 37.85%, 30.51%, 20.00%, 37.32% and 21.75% (Fig. 1b).

### Photosynthetic parameters of rice

**Figure 2:**
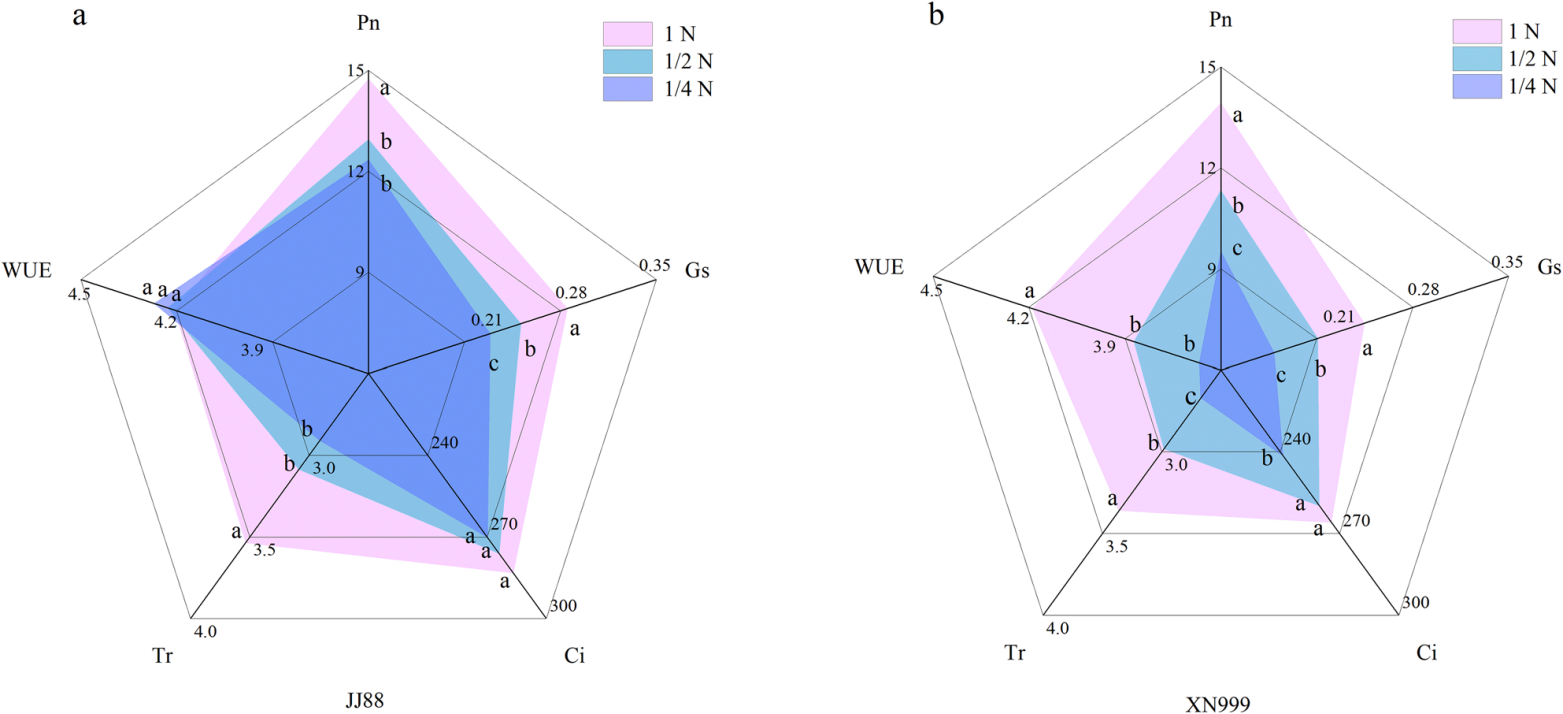
Effect of nitrogen deficiency on photosynthetic parameters (**a, b**) of rice. Each angle of the pentagon showed different traits, Pn—net photosynthetic rate (μmol·m^−2^ s^−1^), Gs—stomatal conductance (mol·m^−2^ s^−1^), Ci—intercellular CO_2_ concentration (μmol·mol^−1^), Tr-transpiration rate (mmol·m^−2^ s^−1^) and WUE water use efficiency (μmol·mol^−1^).

Different nitrogen supply levels have different effects on photosynthetic parameters of rice varieties with different nitrogen efficiencies (Fig. 2). Under 1/2N treatment, Pn, Gs and Tr of JJ88 rice variety significantly (*P* < 0.05) decreased by 12.15%, 14.02% and 12.67%. XN999 rice variety Pn, Gs, Tr and WUE significantly (*P* < 0.05) decreased by 18.61%, 16.84%, 11.26% and 8.48% (Fig. 2a). Under the 1/4N treatment, the Pn, Gs and Tr of the JJ88 rice variety significantly (*P* < 0.05) decreased by 16.27%, 23.33% and 17.68%, and the Pn, Gs, Ci, Tr and WUE of the XN999 rice variety significantly (*P* < 0.05) decreased by 31.59%, 32.78%, 9.18%, 20.53% and 13.95% (Fig. 2b).

### Reactive oxygen metabolism and membrane lipid peroxidation levels

**Figure 3:**
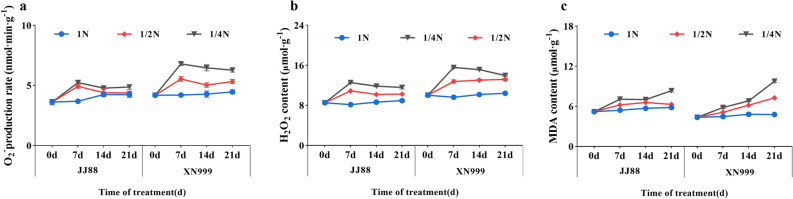
Effects of nitrogen deficiency on rice ${\mathrm{O}}_{2}^{-}$ production rate (**a**), H_2_O_2_ content (**b**) and MDA content (**c**) over days.

Different nitrogen supply levels have different effects on the ${\mathrm{O}}_{2}^{-}$ production rate of leaves of rice varieties with different nitrogen efficiencies (Fig. 3a). Under the 1N treatment, the leaf ${\mathrm{O}}_{2}^{-}$ production rate was relatively stable, and under the 1/2N and 1/4N treatments, it showed a trend of first increasing and then decreasing. The leaf ${\mathrm{O}}_{2}^{-}$ production rate of both rice varieties reached the maximum on the 7th day. After 7 days, the ${\mathrm{O}}_{2}^{-}$ production rates of JJ88 and XN999 rice varieties significantly (*P* < 0.05) increased by 33.60% and 31.90% under 1/2N treatment. Under 1/4N treatment, the ${\mathrm{O}}_{2}^{-}$ production rates of JJ88 and XN999 rice varieties significantly (*P* < 0.05) increased by 42.01% and 61.67%. After 21 days, the ${\mathrm{O}}_{2}^{-}$ production rate of XN999 rice variety under 1/2N treatment significantly(*P* < 0.05) increased by 19.51%.Under 1/4N treatment, the ${\mathrm{O}}_{2}^{-}$ production rate of JJ88 and XN999 rice varieties increased significantly (*P* < 0.05) by 15.13% and 37.5%.

The changing trends of H_2_O_2_ content in leaves of rice varieties with different nitrogen efficiencies were similar after low-nitrogen treatment (Fig. 3b). The changes in H_2_O_2_ content of rice varieties are similar to the changes in ${\mathrm{O}}_{2}^{-}$ production rate. The H_2_O_2_ content increases rapidly and reaches the maximum value at 7 days of treatment. At 7 days of treatment, the H_2_O_2_ production rates of JJ88 and XN999 rice varieties significantly (*P* < 0.05) increased by 33.66% and 47.43% under the 1/2N treatment. Under 1/4N treatment, the H_2_O_2_ production rate of JJ88 and XN999 rice varieties significantly (*P* < 0.05) increased by 54.05% and 61.64%. As low nitrogen treatment continues, the H_2_O_2_ content begins to decrease. After 21 days, the H_2_O_2_ production rates of JJ88 and XN999 rice varieties significantly increased (*P* < 0.05) by 14.78% and 26.83% under 1/2N treatment. Under 1/4N treatment, the H_2_O_2_ production rates of JJ88 and XN999 rice varieties significantly (*P* < 0.05) increased by 29.56% and 34.13%.

The MDA content in the leaves of rice varieties showed an increasing trend with the number of days of low nitrogen treatment (Fig. 3c). The MDA content of JJ88 rice variety increased slowly, while the MDA content of XN999 rice variety increased rapidly. After 21 days, the MDA content of XN999 significantly (*P* < 0.05) increased by 52.2% under 1/2N treatment. Under 1/4N treatment, the MDA content of JJ88 and XN999 rice varieties significantly increased (*P* < 0.05) by 43.13% and 104.4%.

### Antioxidant enzyme activity

**Figure 4:**
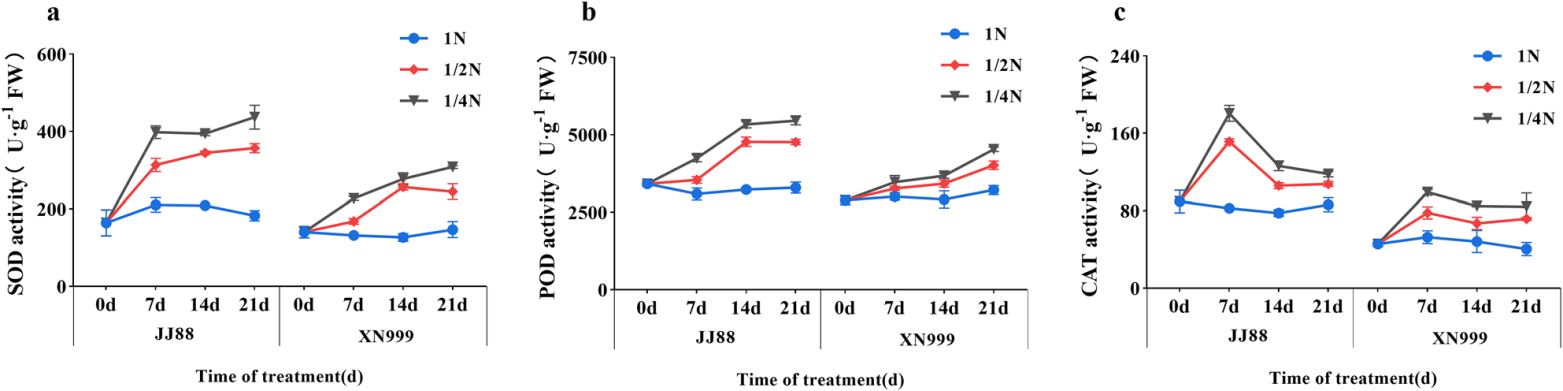
Effects of nitrogen deficiency on rice SOD activity (**a**), POD activity (**b**) and CAT activity (**c**) over days.

The SOD activities of rice varieties with different nitrogen use efficiencies vary greatly (Fig. 4a). Under 1N treatment, the SOD activity of JJ 88 rice variety was slightly higher than that of XN 999 rice variety. After low-nitrogen treatment, SOD activity increased the most from 0 to 7 days. After 7 days of treatment, the SOD activities of JJ88 and XN999 rice varieties under 1/2N treatment significantly increased (*P* < 0.05) by 48.90% and 27.63%, respectively. Under 1/4N treatment, the SOD activities of JJ88 and XN999 rice varieties increased significantly (*P* < 0.05) by 89.20% and 72.17%. After 21 days of treatment, the SOD activities of JJ88 and XN999 rice varieties significantly increased (*P* < 0.05) by 95.17% and 66.67% under 1/2N treatment. Under 1/4N treatment, the SOD activities of JJ88 and XN999 rice varieties increased significantly (*P* < 0.05) by 139% and 110%.

Low nitrogen treatment had large differences in POD activity among rice varieties with different nitrogen use efficiencies (Fig. 4b). The POD activity of XN999 rice variety showed a continuous increasing trend. From 7 to 14 days, the POD activity of the leaves of the two rice varieties increased the most. After 14 days, the POD activity of JJ88 rice variety under 1/2N treatment significantly (*P* < 0.05) increased by 47.63%, and the POD activity of JJ88 and XN999 rice varieties under 1/4N treatment significantly (*P* < 0.05) increased by 64.95% and 26.09%. After 21 days, the POD activity of JJ88 rice variety changed little, and the POD activity of XN999 rice variety increased significantly (*P* < 0.05) by 24.90% under 1/2N treatment. Under 1/4N treatment, the POD activity of XN999 rice variety significantly (*P* < 0.05) increased by 41.08%.

The CAT activity of rice varieties with different nitrogen use efficiencies changed differently after low nitrogen treatment (Fig. 4c). After low-nitrogen treatment, the CAT activity of rice varieties reached the maximum value at 7 days, and the CAT activity decreased and became stable from 7 to 21 days. After 7 days, the CAT activities of JJ88 and XN999 rice varieties under 1/2N treatment significantly (*P* < 0.05) increased by 83.06% and 47.32%, and the CAT activities of JJ88 and XN999 rice varieties under 1/4N treatment significantly (*P* < 0.05) increased by 118.35% and 87.38%.

### ASA–GSH cycle enzyme activity

**Figure 5:**
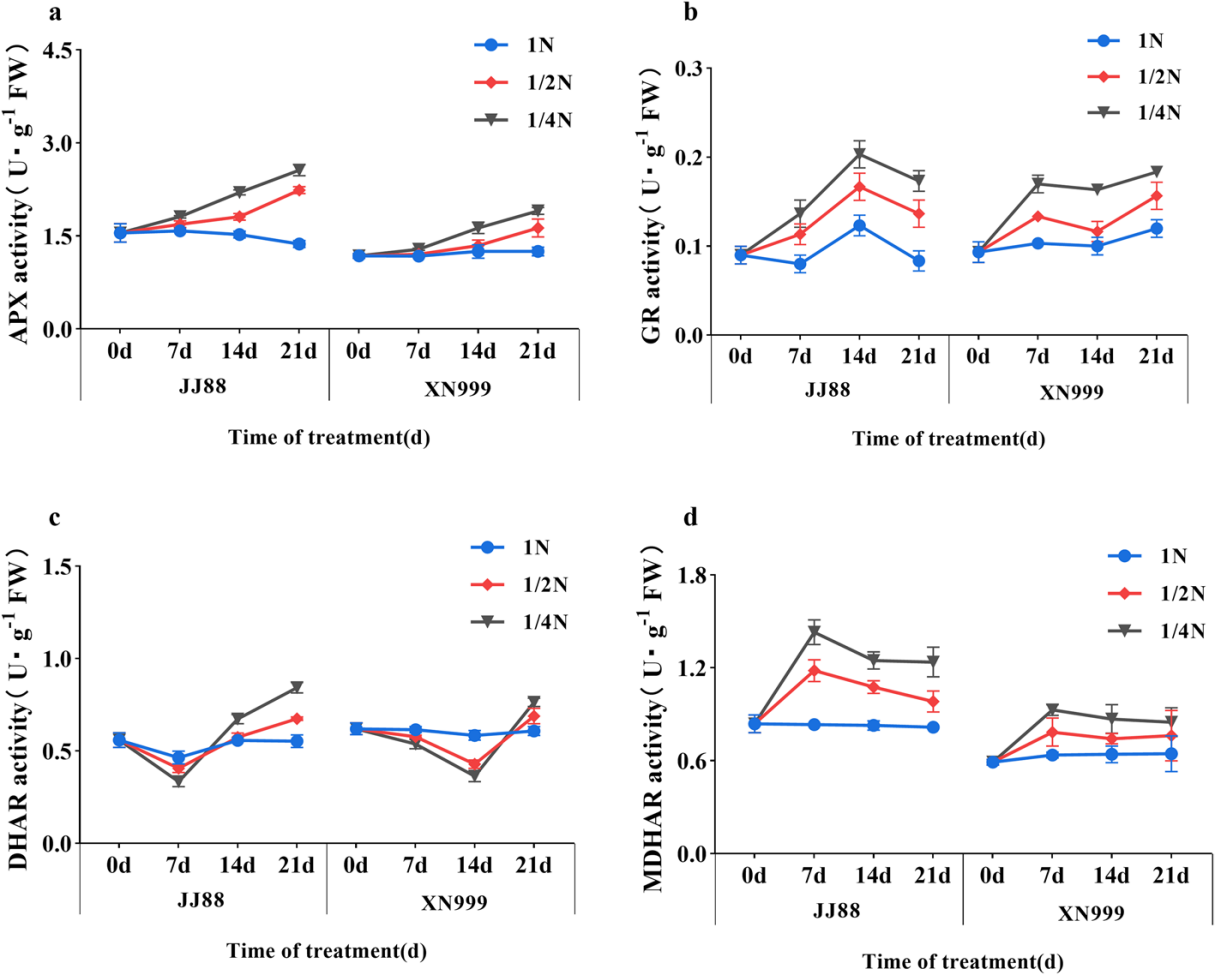
Effects of nitrogen deficiency on rice APX activity (**a**), GR activity (**b**), DHAR activity (**c**) and MDHAR activity (**d**) over days.

The changes in APX, GR, MDHAR and DHAR activities of rice varieties with different nitrogen use efficiencies are different (Fig. 5). The APX activity of rice varieties showed an increasing trend after low nitrogen treatment (Fig. 5a). After 21 days, the APX activities of JJ88 and XN999 rice varieties increased significantly (*P* < 0.05) under 1/2N treatment by 63.48% and 30.48%, and the APX activities of JJ88 and XN999 rice varieties increased significantly (*P* < 0.05) under 1/4N treatment by 87.13% and 52.38%. From 0 to 14 days of low nitrogen treatment, the GR activity of the JJ88 rice variety showed a significant upward trend, and then decreased slightly from 14 to 21 days. During the low nitrogen treatment from 0 to 7 days, the GR activity of the XN999 rice variety showed a significant upward trend, and showed a slight increase from 7 to 21 days (Fig. 5b).

Rice varieties with different nitrogen use efficiencies have different response times under low nitrogen conditions. After 7 days of treatment, the DHAR activities of the JJ88 rice variety under the 1/2N and 1/4N treatments were both lower than those under the 1N treatment. After 21 days of treatment, the DHAR activities of the JJ88 rice variety under the 1/2N and 1/4N treatments were significantly (*P* < 0.05) higher than those under the 1N treatment. The DHAR activity of the XN999 rice variety showed a downward trend from 0 to 14 days of low nitrogen treatment. After 21 days, the DHAR activity of the XN999 rice variety under the 1/4N treatment was significantly (*P* < 0.05) higher than that under the 1N treatment (Fig. 5c). MDHAR and DHAR activities showed opposite trends. After 7 days, the MDHAR activities of JJ88 and XN999 rice varieties under 1/2N treatment significantly (*P* < 0.05) increased by 42.03% and 23.19%; the MDHAR activities of JJ88 and XN999 rice varieties under 1/4N treatment significantly (*P* < 0.05) increased by 71.91% and 45.64% (Fig. 5d).

### Non enzymatic antioxidant content

**Figure 6:**
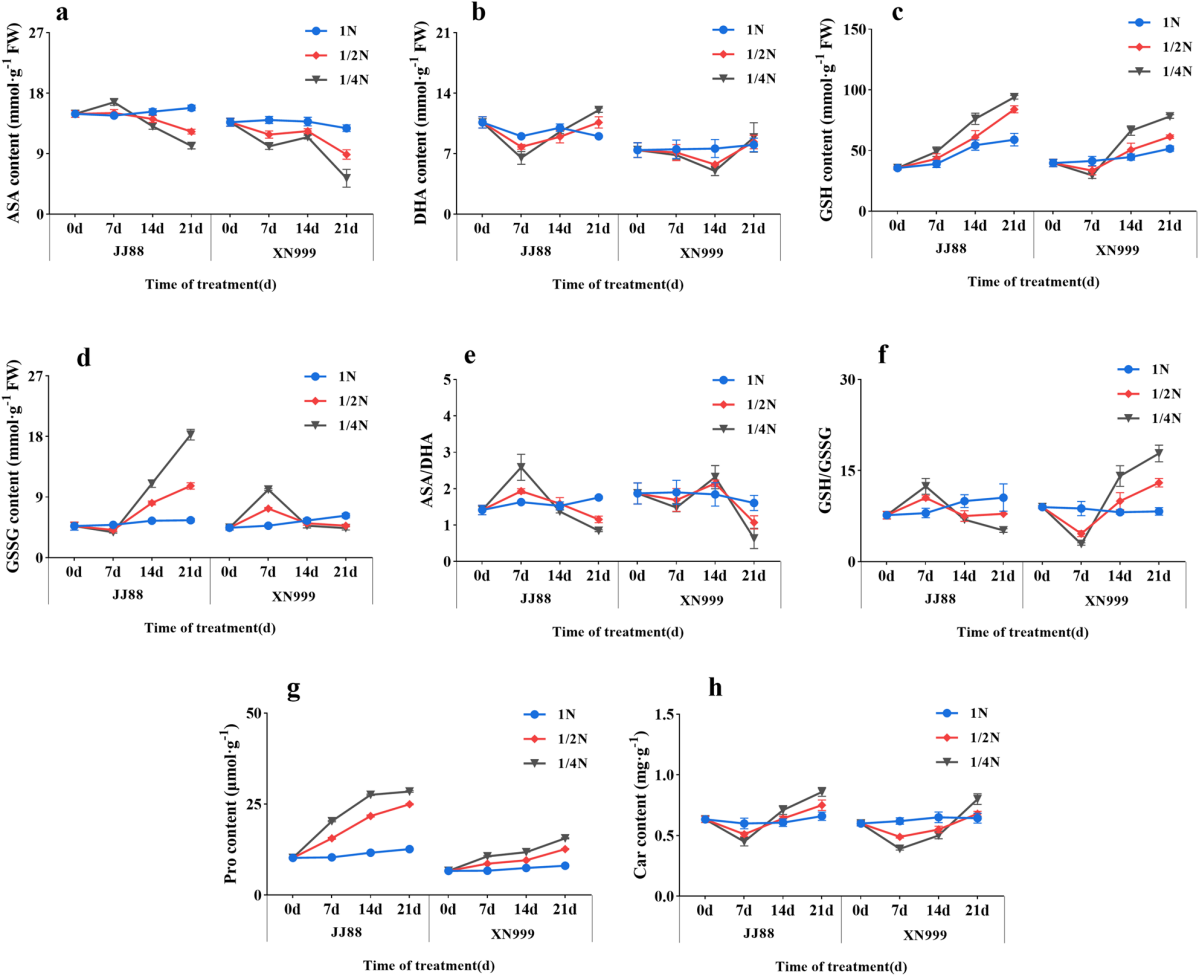
Nitrogen deficiency affects rice ASA content (**a**), DHA content (**b**), ASA/DHA (**c**), GSH content (**d**), GSSG content (**e**), GSH/GSSG (**f**), Pro content (**g**) and Effect of Car content (**h**) with days.

The changes in ASA content of rice varieties with different nitrogen efficiencies were different after low nitrogen treatment (Fig. 6a). After 7 days, the ASA content of JJ88 rice variety under 1/4N treatment significantly (*P* < 0.05) increased by 13.53%. After 21 days, the ASA content of JJ88 rice variety under 1/2N and 1/4N treatments significantly (*P* < 0.05) decreased by 22.39% and 35.83%. After low-nitrogen treatment, the ASA content of XN999 rice variety showed a decreasing trend with days. After 21 days, the ASA content of XN999 rice variety significantly (*P* < 0.05) decreased by 30.62% and 58.32% under 1/2N and 1/4N treatments. After low-nitrogen treatment, the DHA content of JJ88 rice variety showed a downward trend from 0 to 7 days, and the DHA content of XN999 rice variety showed a downward trend from 0 to 14 days (Fig. 6b). After 21 days, the DHA content of JJ88 rice variety under 1/2N and 1/4N treatments significantly (*P* < 0.05) increased by 17.80% and 26.69%. Regarding the changes in ASA/DHA, the two rice varieties showed different change trends, and the response times were also different (Fig. 6e). After 7 days, JJ88 rice variety ASA/DHA was significantly (*P* < 0.05) higher than 1N in 1/2N and 1/4N treatments. After 14 days, the XN999 rice variety ASA/DHA was significantly (*P* < 0.05) higher in the 1/4N treatment than in the 1N treatment.

From 0 to 21 days of low nitrogen treatment, the GSH content of JJ88 rice variety showed an increasing trend, while the GSH content of XN999 rice variety showed a decreasing trend from 0 to 7 days of low nitrogen treatment, and showed an increasing trend from 7 to 21 days (Fig. 6c). The GSSG content of JJ88 rice variety showed a slight decrease from 0 to 7 days of low nitrogen treatment, and then showed an upward trend from 7 to 21 days (Fig. 6d). The changes in GSSG content of JJ88 and XN999 rice varieties showed an opposite trend. Among them, JJ88 and XN999 rice varieties GSH/GSSG also showed opposite trends after low nitrogen treatment (Fig. 6f).

Low nitrogen treatment had an impact on the Pro content of rice varieties (Fig. 6g). The Pro content of JJ88 rice variety increased significantly from 0 to 14 days of low nitrogen treatment. After 14 days, the Pro content of JJ88 rice variety significantly (*P* < 0.05) increased by 86.07% and 136% under 1/2N and 1/4N treatments. The Pro content of XN999 rice variety showed a slow upward trend from 0 to 21 days of low nitrogen treatment. After 21 days, the Pro content of XN999 rice variety significantly (*P* < 0.05) increased by 56.68% and 93.62% under 1/2N and 1/4N treatments.

After low-nitrogen treatment, the Car content in rice leaves showed a trend of first decreasing and then increasing (Fig. 6h). After low-nitrogen treatment, the Car content of JJ88 rice variety accumulated on 14d and 21d, while the Car content of XN999 rice variety only accumulated significantly after 21d. After 21 days, the Car content of JJ88 rice variety under 1/2N and 1/4N treatment was significantly (*P* < 0.05) higher than that of 1N treatment; the Car content of XN999 rice variety under 1/4N treatment was significantly (*P* < 0.05) higher than that of 1N treatment.

### Correlation between active oxygen species, membrane lipid peroxidation levels, antioxidant enzyme activities, ASA–GSH cycle enzymes and non-enzymatic antioxidant contents in leaves under different treatment days

**Figure 7:**
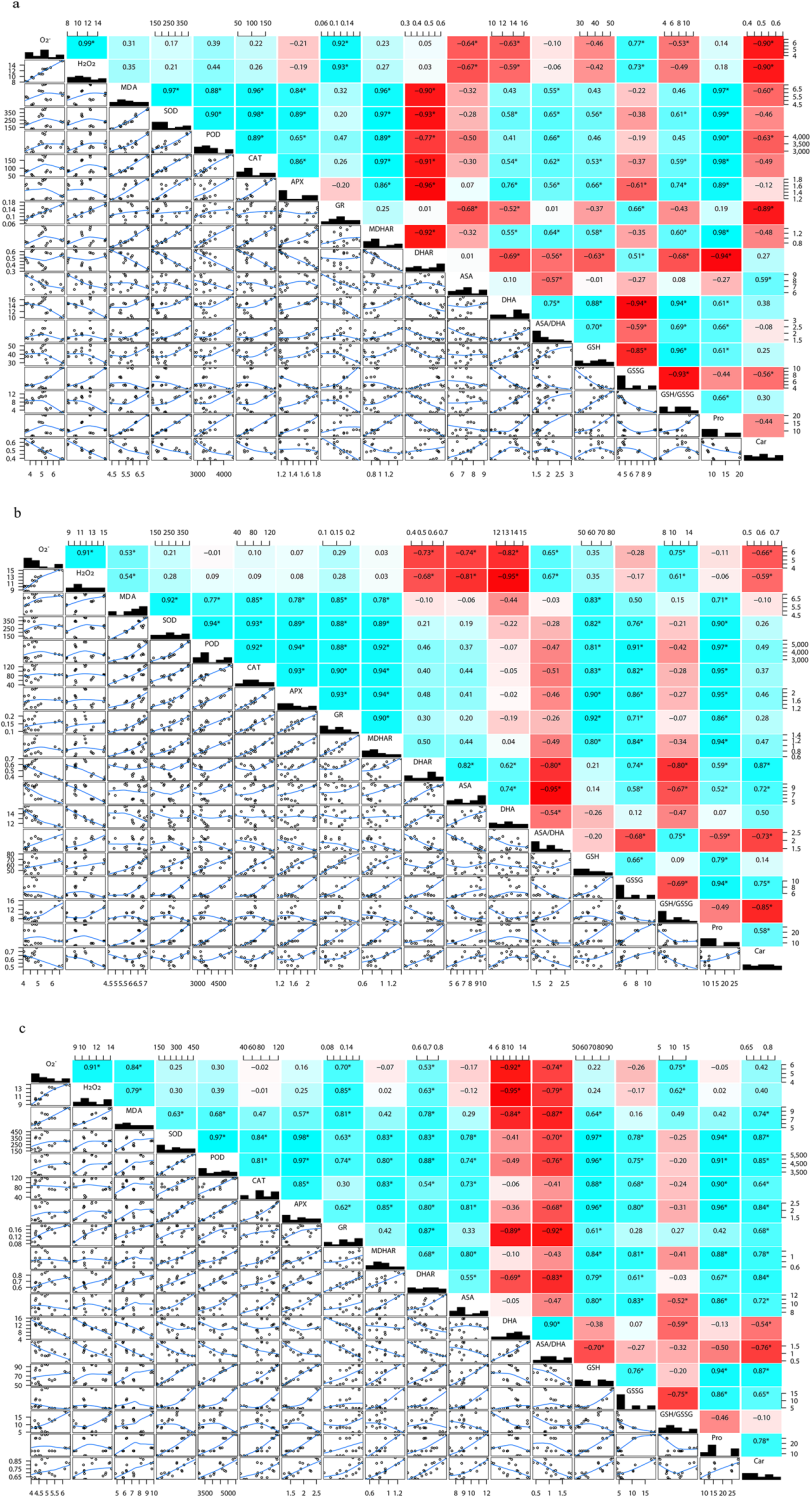
Correlation between reactive oxygen species, membrane lipid peroxidation levels, antioxidant enzyme activities, ASA–GSH cycle enzymes and antioxidant content in different treatments at 7 d (**A**), 14d (**B**) and 21d (**C**). *—significant difference at 0.05 level (*p* < 0.05). The red background of the data in the figure showed negative correlation, while the blue background showed positive correlation.

The correlation analysis of antioxidant data of rice varieties with different treatment days is shown (Fig. 7). After 7, 14 and 21 days of low nitrogen treatment, there was a significant (*P* < 0.05) positive correlation between ${\mathrm{O}}_{2}^{-}$ and H_2_O_2_, a significant (*P* < 0.05) negative correlation between ${\mathrm{O}}_{2}^{-}$ and H_2_O_2_ and DHA and ASA, and a significant (*P* < 0.05) negative correlation between H_2_O_2_ and DHA and ASA. Related, GSSG has a significant (*P* < 0.05) negative correlation with ASA/DHA, GSH/GSSG has a significant (*P* < 0.05) negative correlation with DHAR and GSSG, Car has a significant (*P* < 0.05) negative correlation with ${\mathrm{O}}_{2}^{-}$ and H_2_O_2_, MDA, SOD, Any two of POD, CAT and APX are significantly (*P* < 0.05) positively correlated, MDHAR is significantly (*P* < 0.05) positively correlated with MDA, SOD, POD, CAT and APX, Pro is significantly (*P* < 0.05) positively correlated with SOD, POD, CAT, APX, MDHAR There is a significant (*P* < 0.05) positive correlation with GSH. After 7 days of treatment, DHAR showed a significant (*P* < 0.05) negative correlation with MDA, SOD, POD, CAT, APX and MDHAR. After 14 days, there was a positive correlation. After 21 days, there was a significant (*P* < 0.05) negative correlation. After 7 days of treatment, ASA/DHA showed a significant (*P* < 0.05) positive correlation with MDA, SOD, POD, CAT and APX. After 14 days, there was a negative correlation. After 21 days, there was a significant (*P* < 0.05) positive correlation.

## Discussion

Growth inhibition caused by low nitrogen was an obvious result in previous studies, and our study also showed the same result. Low nitrogen treatment reduced rice plant height, root number, leaf area, aboveground dry weight and underground dry weight. The above-ground and root dry matter accumulation of JJ88 rice variety was higher than that of XN999 rice variety. It has been reported that leaf photosynthesis is largely controlled by the supply and demand of leaf nitrogen content^46^. After the nitrogen supply level decreased, compared with the XN999 rice variety, the JJ88 rice variety had higher Pn and could still maintain relatively large Gs, Ci, Tr, and WUE under low nitrogen levels.This shows that when the nitrogen supply of the JJ88 rice variety is reduced, the growth and development of the plant is less affected, and it maintains a certain photosynthetic capacity, thereby maintaining a certain nitrogen conversion and absorption capacity, and accumulating more dry matter mass. This shows that the JJ88 rice variety can maintain a certain degree of stomata opening under low nitrogen conditions and regulate Ci to affect photosynthesis. This study is consistent with previous research results. Nitrogen-efficient rice varieties have higher leaf area, root density, biomass and Pn under low nitrogen conditions^47,48^. The above results show that compared with XN999, JJ88 rice variety shows stronger growth ability under low nitrogen conditions.

Under stress conditions, excessive reduction in electron transfer in chloroplasts and an overall decrease in photosynthetic electron transfer lead to excessive formation of reactive oxygen species, such as, ${\mathrm{O}}_{2}^{-}$, H_2_O_2_ and OH^−^^25^. Due to the reduction in photosynthesis under low nitrogen conditions, mitochondrial respiration rate increases to compensate for the reduction in ATP synthesis in chloroplasts. High levels of mitochondrial respiration lead to overproduction of ROS by carrying electrons from cytochromes to oxygen^24,49^. This study found that with the persistence of low nitrogen levels, the ${\mathrm{O}}_{2}^{-}$ production rate and H_2_O_2_ content of rice varieties showed an increasing trend. It shows that the intracellular ROS balance is broken and the growth of rice is affected. Since the increase in ${\mathrm{O}}_{2}^{-}$ production rate and H_2_O_2_ content with days in JJ88 rice variety is smaller than that in XN999 rice variety. This shows that low nitrogen conditions cause relatively little damage to the JJ88 rice variety. In previous reports, studies have found that the increase in MDA content in rice seedling leaves is related to toxic substances^50,51^. In this study, we evaluated the toxicity of low nitrogen to rice through the increase in ${\mathrm{O}}_{2}^{-}$ production rate, H_2_O_2_ and MDA content of rice seedlings. Based on these criteria, it was proven that nitrogen deficiency enhanced toxicity in rice, and was relatively less toxic to JJ88 rice variety than to XN999 rice variety.

At present, most studies evaluate the antioxidant capacity of plants based on changes in antioxidant enzyme activity and antioxidant content^52,53^. However, under continuous low-nitrogen conditions, which of rice antioxidant enzymes and antioxidants plays a more important role in plant antioxidant capacity has not been studied. In this study, we found that compared with the 1N treatment, the antioxidant strategies of rice after low-nitrogen treatment did not change, but changed with the extension of low-nitrogen time. The response time and sensitivity of the antioxidant enzyme indicators of the two rice varieties after low nitrogen treatment are different, and the response degrees of the same rice variety to different nitrogen concentrations are also different. Low nitrogen conditions induced SOD, POD and CAT activities in rice varieties. After 7 days of low-nitrogen treatment, the SOD and CAT activities of JJ88 rice variety showed a large increase, and after 14 days, the POD activity showed a large increase. The results show that in the same low-nitrogen environment, the JJ88 rice variety can prevent the surge of ROS by maintaining antioxidant enzyme activity and has a strong ability to maintain the balance of active oxygen metabolism. The previous effects of low nitrogen on the growth and photosynthetic parameters of rice seedlings also verify this view.

The ASA–GSH cycle is the main defense system against ROS. APX and GR are the first and last enzymes of the ASA–GSH cycle respectively, responsible for H_2_O_2_ detoxification^54,55^. The results of this experiment show that with the continuation of low-nitrogen treatment, the JJ88 rice variety has higher APX and GR activities compared with the XN999 rice variety, which is very beneficial for maintaining low ROS content in rice cells. Although the GR activity of JJ88 rice variety decreased slightly after the 21st day of low-nitrogen treatment, it still maintained a higher level compared with the 1N treatment. From the test results, it was also found that the MDHAR activity of the two rice varieties changed in a similar trend under low nitrogen conditions. The activity of MDHAR first increased and then decreased. The early stage of treatment may have caused the stress response of cells to increase its activity, while the decrease in activity in the later stage may have been due to the weakening of the stress response of cells. Although the DHAR activity of the JJ88 rice variety dropped below the 1N level after 7 days, compared with the XN999 rice variety, the decline in DHAR activity of the JJ88 rice variety was relatively small. These results once again show that the JJ88 rice variety exhibits superior physiological characteristics compared with nitrogen-inefficient rice varieties and has stronger low-nitrogen tolerance. The results of this test are consistent with previous studies. Rice with strong resistance usually shows higher antioxidant enzyme activity under stress conditions^56^.

ASA and GSH are the most abundant soluble antioxidants in plants and play a key role in plant resistance to oxidative stress. During the degradation of H_2_O_2_, the change in the ratio of GSH and GSSG is also important in certain redox signaling pathways^57,58^. The ASA–GSH cycle can maintain the oxygen reduction status of cells. Not only can they scavenge reactive oxygen species on their own, but they can also serve as cofactors for oxidative enzymes^54^. Plants show different responses to the ASA–GSH cycle under stress conditions. Changes in GSSG are a sign of plant stress. The GSSG content in rapeseed seedlings also increased similarly after stress^59^. Under high nitrogen conditions, ASA and GSH contents were higher in tomato seedlings, and the ratios of ASA/DHA and GSH/GSSG remained unchanged^60^. Under low nitrogen conditions, the GSH content of soybean varieties with high low nitrogen tolerance increased more than that of soybean varieties with low low nitrogen tolerance^61^. The results of this experiment showed that there were differences in the response of low-nitrogen treatment to ASA–GSH cycle-related indicators of the two rice varieties. On the 7th day of low-nitrogen treatment, the ASA content of JJ88 rice variety increased, while the DHA content decreased, resulting in an increase in ASA/DHA. However, the ASA content of the XN999 rice variety decreased on the 7th day of low-nitrogen treatment, the DHA content remained basically unchanged, and the ASA/DHA ratio decreased. The research results also showed that after 7 days of low nitrogen treatment, the GSH content of JJ88 rice variety increased, the GSSG content decreased, and the GSH/GSSG ratio was higher, but the XN999 rice variety showed the opposite trend. It can be seen that in the early stage of low-nitrogen treatment, the response ability of ASA–GSH cycle related indicators of JJ88 rice variety is higher than that of XN999 rice variety. The higher the GSH/GSSG ratio, the stronger the stress tolerance. The results are consistent with previous studies^59,62^.

Some highly toxic reactive oxygen species cannot be completely eliminated by antioxidant enzymes, so the participation of some small molecule antioxidants is required^29^. Pro is not only an osmotic regulator, but also has the function of quenching ROS^63^. Car is a type of small lipid-soluble molecule, which is of great significance in controlling the accumulation of ROS in the thylakoid membrane system of chloroplasts^25^. In addition, Car plays an important role in preventing photooxidative damage to plants^64^. The results of this experiment showed that during the low-nitrogen treatment, the Pro content of both rice varieties accumulated, indicating an imbalance in the redox state of the rice. Compared with the XN999 rice variety, the JJ88 rice variety accumulated more Pro, indicating that the JJ88 rice variety has a stronger ability to maintain low levels of reactive oxygen species. The Pro content in plants reflects the stress resistance of plants to a certain extent. Varieties with strong stress resistance tend to accumulate more Pro in the body^65^. Low nitrogen treatment promoted the accumulation of Car in rice varieties. After 14 days, the Car content of JJ88 rice variety was higher than that of 1N treatment. After 21 days, the Car content of XN999 rice variety was higher than that of 1N treatment. This shows that the JJ88 rice variety is better than the XN999 rice variety in preventing photo-oxidative damage. The changes in photosynthetic parameters mentioned above verify this view. This study provides theoretical guidance for water and fertilizer management in the seedling stage of rice varieties with different nitrogen efficiencies by analyzing the antioxidant systems of rice varieties with different nitrogen efficiencies. However, further research is needed in subsequent field planting.

## Conclusion

In summary, after low-nitrogen treatment, compared with the XN999 rice variety, the growth (plant height, leaf area, root number, aboveground dry weight and underground dry weight) and photosynthetic parameters (Pn, Gs, Tr, Ci and WUE) of JJ88 rice variety were relatively little affected. The SOD and POD activities of JJ88 rice variety increased with days, and the CAT activity increased rapidly after 7 days of treatment, accumulating relatively little ${\mathrm{O}}_{2}^{-}$, H_2_O_2_, and MDA. The APX activity in the ASA–GSH cycle enzyme of the JJ88 rice variety has a greater increasing trend with days. The GR, DHAR and MDHAR activities have strong responsiveness in the short term. The ASA content in non-enzymatic antioxidant substances shows a downward trend after 14 days of treatment. Car and DHA contents were higher than the 1N treatment level after 14 and 21 days of treatment respectively, and the GSH, GSSG and Pro contents remained at high levels during the entire treatment period. By dynamically regulating antioxidant capacity, JJ88 rice variety has accumulated relatively less ROS, making it exhibit low nitrogen tolerance.

## Data Availability

The authors confirm that the data supporting the findings of this study are available within the article.
